# Altered resting-state functional connectivity and dynamic network properties in cognitive impairment: an independent component and dominant-coactivation pattern analyses study

**DOI:** 10.3389/fnagi.2024.1362613

**Published:** 2024-03-18

**Authors:** Maurizio Bergamino, Anna Burke, Marwan N. Sabbagh, Richard J. Caselli, Leslie C. Baxter, Ashley M. Stokes

**Affiliations:** ^1^Division of Neuroimaging Research, Barrow Neurological Institute, Phoenix, OK, United States; ^2^Division of Neurology, Barrow Neurological Institute, Phoenix, OK, United States; ^3^Department of Neuropsychology, Mayo Clinic Arizona, Phoenix, AZ, United States; ^4^Department of Neurology, Mayo Clinic Arizona, Phoenix, AZ, United States

**Keywords:** cognitive impairment, resting-state functional connectivity, independent component analysis, dominant-coactivation pattern analysis, dementia

## Abstract

**Introduction:**

Cognitive impairment (CI) due to Alzheimer’s disease (AD) encompasses a decline in cognitive abilities and can significantly impact an individual’s quality of life. Early detection and intervention are crucial in managing CI, both in the preclinical and prodromal stages of AD prior to dementia.

**Methods:**

In this preliminary study, we investigated differences in resting-state functional connectivity and dynamic network properties between 23 individual with CI due to AD based on clinical assessment and 15 healthy controls (HC) using Independent Component Analysis (ICA) and Dominant-Coactivation Pattern (d-CAP) analysis. The cognitive status of the two groups was also compared, and correlations between cognitive scores and d-CAP switching probability were examined.

**Results:**

Results showed comparable numbers of d-CAPs in the Default Mode Network (DMN), Executive Control Network (ECN), and Frontoparietal Network (FPN) between HC and CI groups. However, the Visual Network (VN) exhibited fewer d-CAPs in the CI group, suggesting altered dynamic properties of this network for the CI group. Additionally, ICA revealed significant connectivity differences for all networks. Spatial maps and effect size analyses indicated increased coactivation and more synchronized activity within the DMN in HC compared to CI. Furthermore, reduced switching probabilities were observed for the CI group in DMN, VN, and FPN networks, indicating less dynamic and flexible functional interactions.

**Discussion:**

The findings highlight altered connectivity patterns within the DMN, VN, ECN, and FPN, suggesting the involvement of multiple functional networks in CI. Understanding these brain processes may contribute to developing targeted diagnostic and therapeutic strategies for CI due to AD.

## 1 Introduction

Cognitive impairment (CI) is a decline or impairment of cognitive abilities, encompassing memory, attention, language, and perception. In aging populations, CI can be attributed to many different etiologies, including neurodegenerative diseases such as Alzheimer’s disease (AD), vascular disease, and even neuropsychiatric disorders. Of these, AD is the most common form of dementia, currently affecting 5.7 million people in the United States (Weiner et al., 2015, 2017). On the other hand, mild cognitive impairment (MCI) refers to the stage between the normal age-related decline of memory and thinking skills and more severe decline seen in dementia (Petersen et al., 1999). In many cases, MCI represents the prodromal stage of major neurocognitive disorders, such as AD (Sacuiu et al., 2016).

Different magnetic resonance imaging (MRI) techniques have been used to investigate brain changes in people with CI. For instance, resting-state functional MRI (rs-fMRI; Biswal et al., 1997) is an MRI technique for the early detection of dementia (Vemuri et al., 2012; Matthews and Hampshire, 2016). By examining changes in brain network functional connectivity, particularly within the default mode network (DMN), rs-fMRI has been utilized to identify early signs of AD (He et al., 2007). Notably, various fMRI techniques have been proposed to analyze functional connectivity, including sliding-window (Chang and Glover, 2010), temporal independent component analysis (ICA; Smith et al., 2012), and quasi-periodic pattern (Thompson et al., 2014) methods.

ICA is a data-driven method that aims to identify underlying brain networks within fMRI data (Hyvärinen and Oja, 2000). ICA has been widely utilized in AD and MCI studies to explore the underlying brain alterations associated with these conditions (Greicius et al., 2004; Li et al., 2012; Wang et al., 2012). Indeed, ICA analysis can detect abnormal connectivity patterns or dysfunction in key brain networks affected by AD, such as the DMN (Yıldırım and Soncu Büyükişcan, 2019) or the salience network (Zhang et al., 2020). In addition, longitudinal studies utilizing ICA have revealed alterations in functional connectivity patterns over time, providing insights into their progression (Abubakar et al., 2012).

However, in recent years, there has been growing interest in studying the dynamics of the brain’s intrinsic networks using co-activation pattern (CAP) analysis. Liu and Duyn introduced the CAP approach to track functional connectivity variations within each time frame (Liu and Duyn, 2013). In the CAP method, the fMRI volumes of the entire brain at time points with significant fMRI signals are temporally clustered using k-means into a predetermined number of CAPs, which reflect the dynamic behavior of a particular resting-state network. This method offers a notable advantage by concentrating on individual time frames, eliminating the need for an extensive number of input time points compared to other fMRI analysis methods. Additionally, CAP analysis establishes a more direct relationship between voxels, in contrast, for instance, to the correlation-based sliding window method. Importantly, the versatility of CAP analysis allows for its extension to whole-brain analysis, incorporating the entire fMRI volume into temporal clustering.

In 2018, Zhuang et al. proposed the dominant-CAP (d-CAP) method (Zhuang et al., 2018), which aggregates CAPs from multiple clustering runs. This method has been applied in individuals with Parkinson’s disease and healthy controls (HCs), providing quantitative information on network temporal dynamics in both groups (Zhuang et al., 2018). In addition to the spatial d-CAPS, this novel analysis yields the number of d-CAPs, the temporal fraction and spatial consistency of each d-CAP, and the subject-specific switching probability among all d-CAPs for different groups. These measures can be used to compare network dynamics between groups.

In this preliminary study, following the methodology introduced by Zhuang et al. (2018), we synthesized a set of d-CAPs by combining CAPs obtained from multiple clustering runs for a group of CI and HCs in the default mode network (DMN), visual network (VN), executive control network (ECN), and frontoparietal network (FPN). Additionally, the same networks were analyzed by the conventional ICA method. One key benefit of d-CAP analysis is its ability to focus on individual time frames, reducing the need for many input time points compared to other methods. In addition, d-CAP analysis captures a more direct voxel relationship than the correlation-based sliding window method (Liu and Duyn, 2013). It also allows analysis of the whole brain in the entire volume of fMRI for temporal clustering (Liu et al., 2013). We aimed to apply this innovative approach to examine the temporal dynamics of resting-state brain networks in dementia populations.

## 2 Methods

### 2.1 Subjects

This study was performed in accordance with the local Institutional Review Board. All participants gave written, informed consent in this HIPAA-compliant study.

The study included a total of 15 HC (9 females; mean age [SD]: 74.3 [6.5] years) and 23 individuals with CI (14 females; mean age [SD]: 76.9 [6.8] years). The CI group was comprised of 13 AD patients in the stage of dementia (7 females; mean age [SD]: 77.8 [8.2] years) and 10 MCI patients due to AD (7 females; mean age [SD]: 75.8 [4.8] years). All individuals with CI were referred from local neurology clinics or selected from the Arizona Alzheimer’s Disease Center (AADC) database (15 individuals from neurology clinics and 8 individuals from AADC). The inclusion of subjects in each cohort was based on clinical diagnostic criteria determined by a practicing neurologist (Albert et al., 2011; McKhann et al., 2011). Prior to the MRI scan, all participants underwent cognitive assessments, including the Montreal Cognitive Assessment (MoCA; Nasreddine et al., 2005), and Clock-Draw test (Sunderland et al., 1989). The complete subject characteristics are summarized in Table 1.

**Table 1:** The complete subject characteristics for this study.

Group	N (F)	age (S.D) years	motion (mean displacement) mm	MoCA	Clock-draw	
HC	15 (9)	74.3 (6.5)	0.30 (0.15)	26.00 (2.44)	1.82 (0.98)	
CI	23 (14)	76.9 (6.8)	0.29 (0.10)	15.65 (6.26)	3.43 (1.12)	
Shapiro–Wilk		W = 0.97; *p* = 0.41	W = 0.84; *p* < 0.001	W = 0.92; *p* < 0.001	W = 0.91; *p* = 0.010	
t-test:		t = 1.17; *p* = 0.25	-	-	-	
Mann–Whitney:		-	U = 199; *p* = 0.438	U = 16; *p* < 0.001	U = 216; *p* < 0.001	
CI group						
AD	13 (7)	77.8 (8.2)	0.29 (0.07)	12.69 (5.51)	3.85 (0.99)	
MCI	10 (7)	75.8 (4.8)	0.30 (0.13)	19.50 (5.10)	2.90 (1.10)	
t-test:		t = 0.76; *p* = 0.46	-	-	-	
Mann–Whitney:		-	U = 70; *p* = 0.784	U = 21.5; *p = 0.007*	U = 91; *p* = 0.088	
MoCA domains (for CI)						
	Executive functions	Attention & concentration	Memory	Language	Visuospatial skills	Orientation
AD	0.83 (1.03)	1.50 (1.51)	0.00 (0.00)	1.50 (1.68)	0.75 (0.73)	0.92 (1.24)
MCI	1.20 (1.75)	1.70 (2.41)	0.90 (1.62)	1.60 (2.12)	1.10 (1.52)	2.10 (2.76)
Mann–Whitney:	U = 59; *p* = 0.971	U = 62.5; *p* = 0.888	U = 36; *p* = 0.021	U = 59; *p* = 0.971	U = 59; *p* = 0.972	U = 54.5; *p* = 0.718

SD, Standard Deviation; MoCA, Montreal Cognitive Assessment. The Shapiro–Wilk test was used to assess the normality of the data sample (Shapiro and Wilk, 1965).

### 2.2 MRI acquisition

MRI data were acquired at 3 T (Ingenia, Philips, Best, Netherlands) with a dedicated 32-channel head coil. Standard T1-weighted (T1-w) anatomical images were acquired using a 3D magnetization-prepared rapid acquisition gradient echo (MP-RAGE) sequence with the following acquisition parameters: repetition time / echo time (TR/TE), 6.7/3.104 msec; acquisition matrix, 256 × 256; voxel size, 1.06 × 1.06 mm; slice thickness, 1.2 mm; 170 sagittal slices; flip angle = 9^0^. rs-fMRI was acquired using an echo-planar imaging (EPI) acquisition with TR/TE, 3.000/30.0 msec acquisition matrix, 215 × 215; voxel size, 2.68 × 2.68 mm; slice thickness, 2.68 mm; flip angle = 80^0^; 140 volumes.

### 2.3 Rs-fMRI pre-processing

The rs-fMRI datasets were converted to NIFTI format using *dcm2niix*.^1^ To ensure a signal steady state, the first three-time frames were removed from each dataset (*3dTcat*, AFNI)^2^ and temporal de-spiking was applied using *3dDespike* (AFNI).

Image distortions can arise from head movements during data acquisition, potentially compromising data quality and impeding accurate interpretation of results. Even minor head movements can introduce variations in measured blood oxygenation levels, contributing to increased data variability. Therefore, implementing data preprocessing to minimize head movement is essential to improve the final results. In this study, motion parameters were estimated, and realignment of each time series was performed using *3dvolreg* (AFNI). Following visual inspection of the estimated motion parameters, participants with motion exceeding thresholds (>3 mm translation, >3 degrees rotation) were excluded from the study. These criteria were established in alignment with voxel size and considerations for the anticipated spatial resolution of BOLD responses, accounting for inherent variability in brain anatomy across subjects.

A brain mask was generated by *bet* (Smith, 2002; FSL)^3^ based on the average volume of each motion-corrected time series. Each brain-extracted rs-fMRI was then coregistered to the corresponding relative MPRAGE (also brain extracted by *bet*) and spatially normalized to the standard MNI template (2 mm) using a 12-parameter affine transformation and mutual-information cost function (FLIRT; Jenkinson and Smith, 2001; FSL). Additionally, the data were resampled to isotropic resolution with a Gaussian kernel of FWHM = 4 mm (FSL). Voxel time-courses were bandpass filtered using AFNI (0.008 Hz < *f* < 0.1 Hz) to highlight low-frequency correlations during resting state.

### 2.4 ICA processing

The rs-fMRI datasets of the two groups were separately used for group ICA analysis using the Multivariate Exploratory Linear Optimized Decomposition into Independent Components (MELODIC) program (version 3.15; Beckmann and Smith, 2004), which is part of the FSL package. The analysis option of multi-session temporal concatenation was chosen to extract common spatial patterns without assuming a consistent temporal response across subjects. The resulting independent components (IC) maps were thresholded using a mixture model and alternative hypothesis testing approach. The threshold parameter was set to 0.5 to achieve an equal weight in false positives and false negatives (Woolrich et al., 2005).

Examining various component options and understanding the reasoning behind their selection is crucial for gaining insights into the robustness of identified resting components. In this study, we analyzed all MELODIC components for each network, averaging them when multiple components were present, to ensure a more resilient identification of the resting network. Furthermore, we scrutinized three key aspects for each component: the spatial map, the time course, and the power spectrum of the time course. Following this procedure, the DMN, VN, ECN, and FPN were the specific networks analyzed in the study.

Dual regression, through the *dual_regression* tool in FSL, was employed to estimate customized group-level spatial maps and time courses for each participant’s 4D data across the four networks of interest (Nickerson et al., 2017). The process involved two steps. In the first stage, the group spatial maps were regressed onto each participant’s 4D dataset, producing a set of time courses. In the second stage, these time courses were further regressed onto the same 4D dataset, yielding subject-specific spatial maps. Consequently, this analysis generated a component-time course and spatial map for each subject and each component.

### 2.5 D-CAPs processing

**Figure 1:**
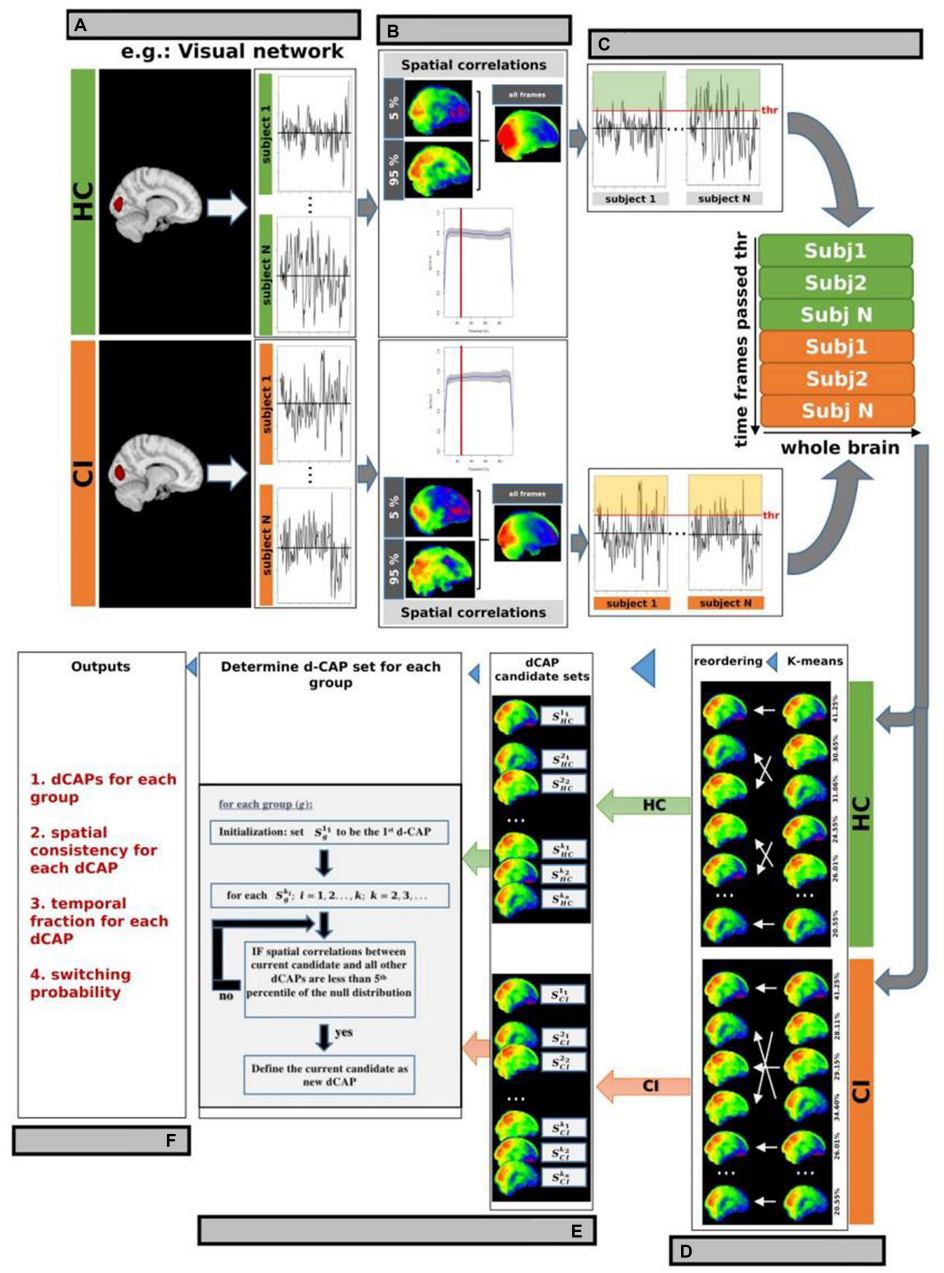
d-CAP processing for the Visual Network (VN; as an example). **(A)** Conventional CAP analysis used a single seed region of interest (ROI) to identify fMRI volumes associated with a specific resting-state network in each subject and group. The spatial correlation between the average of selected time frames and the network-seeded correlation is shown in panel **(B)**. The gray background represents regions within ±1 SD across study’s participants. **(C)** A threshold of 25% was chosen based on the spatial correlation (panel **B**), and network-associated time points were defined when the averaged seed signal intensities surpassed this threshold. Whole-brain fMRI signals at these time points were identified and temporally concatenated for each subject in both HC and CI groups. **(D)** Temporal clustering was performed on the concatenated time frames from both groups to ensure correspondence between CAPs from the HC and CI groups. **(E)** d-CAPs set were determined for both group. More information about the algorithm can be found in Zhuang et al. **(F)** From d-CAPs different outputs can be obtained for statistical analysis.

Briefly, before performing d-CAP processing, voxel signals of the rs-fMRI data, in MNI standard space, were normalized by demeaning and dividing by the temporal SD. Conventional CAP analysis utilizes a single seed region of interest (ROI) to identify fMRI volumes associated with a specific resting-state network, such as the VN shown in Figure 1A. In this study, we generated automated 3 mm sphere ROIs within each network. Subsequently, the mean signal intensity within each ROI was computed and averaged, yielding a singular time-course per subject for each network. The spatial correlation between the average of selected time frames and the network-seeded correlation map increased with more frames by lowering the threshold. As a result, the spatial correlation led to a threshold of 25% (Figure 1B), and network-associated time points were included when the averaged seed signal intensities exceeded the chosen threshold. Whole-brain fMRI signals at these time points were identified for each subject in both HC and CI groups and were temporally concatenated, as depicted in Figure 1C.

Temporal clustering was performed on the concatenated time frames from both groups to ensure correspondence between CAPs from the HC and CI groups. K-means clustering was used with multiple cluster numbers (*k* = 1, 2, 3; …). Group-specific CAP sets ($S^{k_{i1}}$, $S^{k_{i2}}$, …) were computed by averaging spatial maps corresponding to time frames assigned to each cluster *k*_i_ from the HC and CI groups (Figure 1D).

In this study, a separate d-CAP set was calculated for each group, giving equal consideration to every CAP generated from different k-means runs to avoid bias from a single run. The final d-CAP set integrated clustering results from multiple k-means runs, capturing dynamic structures within network-associated time frames. After determining each group’s final d-CAP set, fMRI signals at network-associated time points were assigned to different clusters based on their spatial similarities to the d-CAPs. Spatial correlations were calculated between each network-associated time frame and every d-CAP to evaluate spatial resemblance. This information was utilized to assign the specific cluster (Figure 1E).

From d-CAPs analysis, several metrics can be obtained (Figure 1F):

1. Number of d-CAPs: As the computation of d-CAP sets for each group is data-driven, the number of d-CAPs inherently reflects the network-associated dynamics within each group. Consequently, a smaller number of d-CAPs for a group signifies a less dynamic network.
2. Spatial consistency: To assess spatial consistency, the correlation between an individual network-associated time frame and the corresponding d-CAP map is calculated for each d-CAP cluster. These correlations are then averaged to determine the level of spatial consistency. A higher temporal fraction, combined with a more stable spatial consistency, indicates a resting-state network with reduced dynamics.
3. Temporal fraction: The temporal fraction (TF) is a metric utilized to quantify the duration for which a specific network remains within a given d-CAP throughout the scan. An imbalanced distribution of temporal occurrences suggests that a particular group spends more time within one or more d-CAPs, indicating reduced network dynamics. TF is defined for each d-CAP as follows:
  $$TF_{j}=\frac{\text{number of time}-\text{frames assigned to}\;{\text{dCAP}}^{j}}{\text{number of networks associated time}-\text{frames}}$$

with *j* = 1,2,3,…., number of d-CAPs.

Switching probability: the switching probability (SP) can be also computed for each subject to measure the dynamics of d-CAPs associated with each resting-state network. As previously mentioned, network-associated time frames are assigned to respective d-CAPs. If two consecutive time frames are assigned to different d-CAPs, it is considered a d-CAP switch. A reduced switching probability in one group reflects a less dynamic network. The SP for a subject is defined as follows:


$$SP_{s}=\frac{\text{number of}\;d-\text{CAP}\;\text{switches}}{\text{number of networks associated time}-\text{frames for subj}\;s}$$

with *s* = 1,2,3,…., number of subjects.

### 2.6 Statistical analysis

The mean and SD are provided for age and cognitive assessment scores. Age differences were examined using the Student t-test (Shapiro–Wilk: W = 0.97; *p* = 0.41), while differences in cognitive test scores were analyzed using the Mann–Whitney test (Shapiro–Wilk: *p* < 0.001 for all tests). Differences in motion during the rs-fMRI acquisition were evaluated by the Mann–Whitney test (Shapiro–Wilk: *p* < 0.001). Statistical significance was set at *p* < 0.05, with a power analysis of 0.76 (α=0.05; effect-size = 0.80).

In the dual-regression procedure (ICA), the group-level statistical inference was performed through a linear model at the voxel level, with motion, age and sex as covariates, using an in-house R script (version 4.3.1) and RStudio (version 2023.06.0). *p*-values were corrected using the False Discovery Rate (FDR) method to address multiple comparisons. Probabilistic threshold-free cluster enhancement (pTFCE; Spisák et al., 2019) was used to control for multiple comparison correction on each component separately with corrected probability of 0.05 determined as the significance threshold. Furthermore, given our analysis of four distinct networks, we applied the Bonferroni correction to adjust the final *p*-values. For ICA, effect-size and associated confidence interval were calculated by Cohen-*d* with R and RStudio (large effect for *d* > ±0.80).

d-CAPs were processed using an in-house R and RStudio script, employing the algorithm described in Zhuang et al. (2018). In this analysis, we explored a range of cluster numbers in k-means, spanning from 2 to 20. To mitigate the instability of single-trial k-means outcomes, we repeated the clustering process 100 times. The clustering result with the lowest within-cluster sum of point-to-centroid distances was selected as the optimal outcome.

To investigate differences in the switching probability of each network, a Student t-test was conducted with age and gender as covariates. FDR correction was used to address multiple comparisons. Differences in the temporal fraction of the 1^st^ d-CAPs for the networks with the same number of d-CAPs was also evaluated by Student t-test with age and gender as covariates, followed by FDR correction.

Correlations between switching probabilities and cognitive scores were evaluated by a linear model, with age and sex as covariates, by R and RStudio. The *p*-values were corrected by FDR. Effect size for correlations was evaluated by Spearman’s rank correlation coefficient (ρ).

The resulting clusters were labeled according to the automated anatomical labeling atlas (AAL; Tzourio-Mazoyer et al., 2002).

## 3 Results

No statistical differences were found for age (*t* = 1.17; *p* = 0.25) across the two groups. On the other hand, differences between HC and CI were observed for all cognitive tests (MoCA: U = 16; *p* < 0.001; Clock-Draw: U = 216; *p* < 0.001). Additionally, no differences between groups were found for the motion (mean displacement) during the rs-fMRI acquisition (U = 199; *p* = 0.438).

For the CI group (comparison between AD and MCI), we did not find any statistical differences in age (*t* = 0.76; *p* = 0.46), motion (mean displacement; U = 70; *p* = 0.784), or the clock-draw test (U = 91; *p* = 0.088). However, differences between AD and MCI were found for the MoCA (U = 21.5; *p* = 0.007). Analyzing the different MoCA domains, differences between AD and MCI were seen only in the memory domain (U = 36; *p* = 0.021; see Table 1). All subjects were included in the final analysis, and for all subsequent analyses, the MCI and AD groups were combined into the CI group.

Table 2 displays the number of d-CAPs identified in both the HC and CI groups for the resting-state networks utilized in the CAP group analysis. We observed an equal number of d-CAPs for the DMN, ECN, and FPN in both groups. However, our analysis detected a lower number of d-CAPs for the VN for the CI group compared to the HC group.

**Table 2:** Number of d-CAPs for both groups for resting-state networks used in the CAP group analysis.

Network	HC	CI
DMN	3	3
VN	5	3
ECN	3	3
FPN	2	2

### 3.1 Default mode network

**Figure 2:**
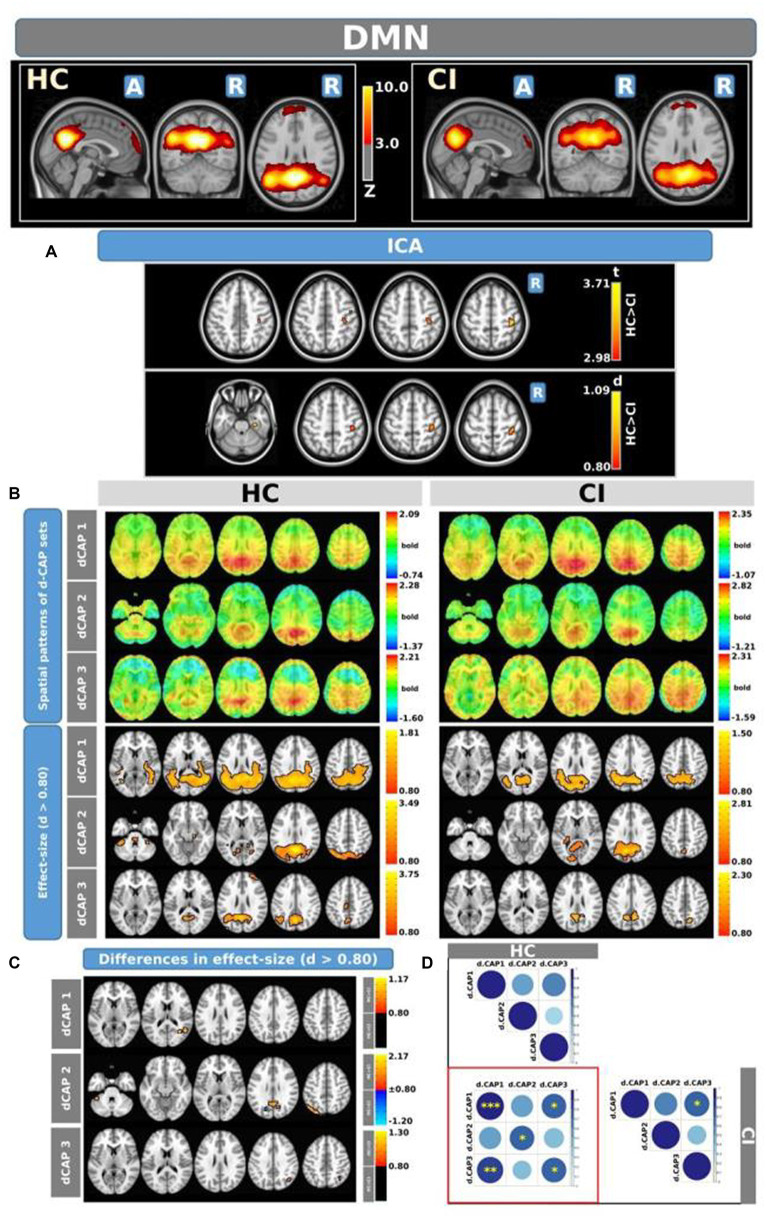
For the DMN: Panel **(A)** displays the ICA results for both t-test and effect-size, while panel **(B)** shows the spatial patterns of the d-CAP sets in both the HC and CI groups. In panel **(C)**, effect size maps for each d-CAP in each group are presented. Additionally, panel **(D)** exhibits the spatial correlation matrices between the d-CAPs, denoted by asterisks (*) to indicate spatial similarities: *for *r* > 0.70, **for *r* > 0.80, and ***for *r* > 0.90.

The Z-values, from the dual-regression procedure, for the DMN are shown in Figure 2. Panel A shows the statistical differences between HC and CI for ICA analysis. Significantly lower connectivity was found in the CI group than HC in the right precentral (<t > =2.979) and right postcentral gyrus (<t > =3.096). The complete results, with effect sizes and confidence intervals, are reported in Table 3A.

**Table 3:** Complete results for DMN using **(A)** ICA and **(B)** d-CAP analysis.

DMN								
(A)								
ICA (t-test clusters)								
	HC > CI (FWE < 0.05 + Bonferroni)							
AAL	Vol (%)	t	*d*	Conf. I				
Precentral R	0.68	2.981	0.831	[0.147–1.510]				
Postcentral R	3.55	3.116	0.923	[0.220–1.607]				
(B)								
d-CAPs								
	d-CAP 1		d-CAP 2				d-CAP 3	
	HC > CI		HC > CI		HC < CI		HC > CI	
AAL	Vol (%)	<Δ*d*>	Vol (%)	<Δ*d*>	Vol (%)	<Δ*d*>	Vol (%)	<Δ*d*>
Precentral L	-	-	0.82	1.046	-	-	-	-
Precentral R	-	-	0.35	1.003	-	-	-	-
Cingulum Post L	-	-	0.35	0.962	-	-	-	-
Cingulum Post R	-	-	2.11	1.351	-	-	-	-
Calcarine R	-	-	0.62	1.118	-	-	-	-
Cuneus L	-	-	0.80	1.083	0.22	−0.951	-	-
Cuneus R	-	-	2.49	1.159	-	-	-	-
Occipital Sup L	-	-	2.19	1.076	1.54	−0.994	-	-
Occipital Sup R	-	-	-	-	-	-	0.39	0.904
Occipital Mid L	-	-	1.01	1.049	-	-	-	-
Occipital Mid R	-	-	-	-	-	-	0.49	0.925
Postcentral L	-	-	6.08	0.997	-	-	-	-
Postcentral R	-	-	1.56	1.035	-	-	-	-
Parietal Sup L	-	-	26.71	1.184	-	-	-	-
Parietal Sup R	-	-	11.43	1.160	-	-	-	-
Parietal Inf L	-	-	10.64	1.222	-	-	-	-
Angular L	-	-	19.28	1.179	-	-	-	-
Angular R	0.40	0.847	-	-	-	-	3.09	0.922
Precuneus L	-	-	5.10	1.071	0.35	−1.020	-	-
Precuneus R	-	-	4.27	1.205	-	-	-	-
Paracentral Lobule L	-	-	2.19	0.942	-	-	-	-
Temporal Sup R	0.25	0.894	-	-	-	-	-	-
Temporal Mid R	2.66	0.935	-	-	-	-	-	-

AAL, automated anatomical labeling atlas; Vol (%), volume in percent covered from the cluster in the relative AAL area; d, Cohen-*d* effect-size; Conf.I, confidence interval for the effect-size; <Δ*d*>, differences in effect-size (thresholded at ±0.80).

Panel B displays the spatial patterns of the blood oxygen level-dependent (BOLD) signal for the final d-CAP sets of the DMN. For this network, for both groups, we found 3 d-CAPs. Each d-CAP’s spatial map was also converted to effect size (Cohen’s *d*) maps, which were thresholded at d = ±0.80, representing the large effect size. For each d-CAP, between-group differences in Cohen’s *d* maps at *d* ≥ ±0.80 are reported in panel C and Table 3B. Increased coactivation (indicated by large effect size) for all d-CAPs was observed in the HC group. On the other hand, for d-CAP-2, small clusters of larger activity were observed in CI groups inside the left cuneus and precuneus and inside the left superior occipital gyrus. Spatial similarities between d-CAPs are represented by correlation matrices and are shown in panel D. The between-group d-CAP spatial similarities are shown inside the ‘red’ box. For this network, high spatial similarity was found for d-CAP-1 between HC and CI (*r* > 0.90) and for d-CAP-1 (HC) and d-CAP-3 (CI; *r* > 0.80).

### 3.2 Visual network

**Figure 3:**
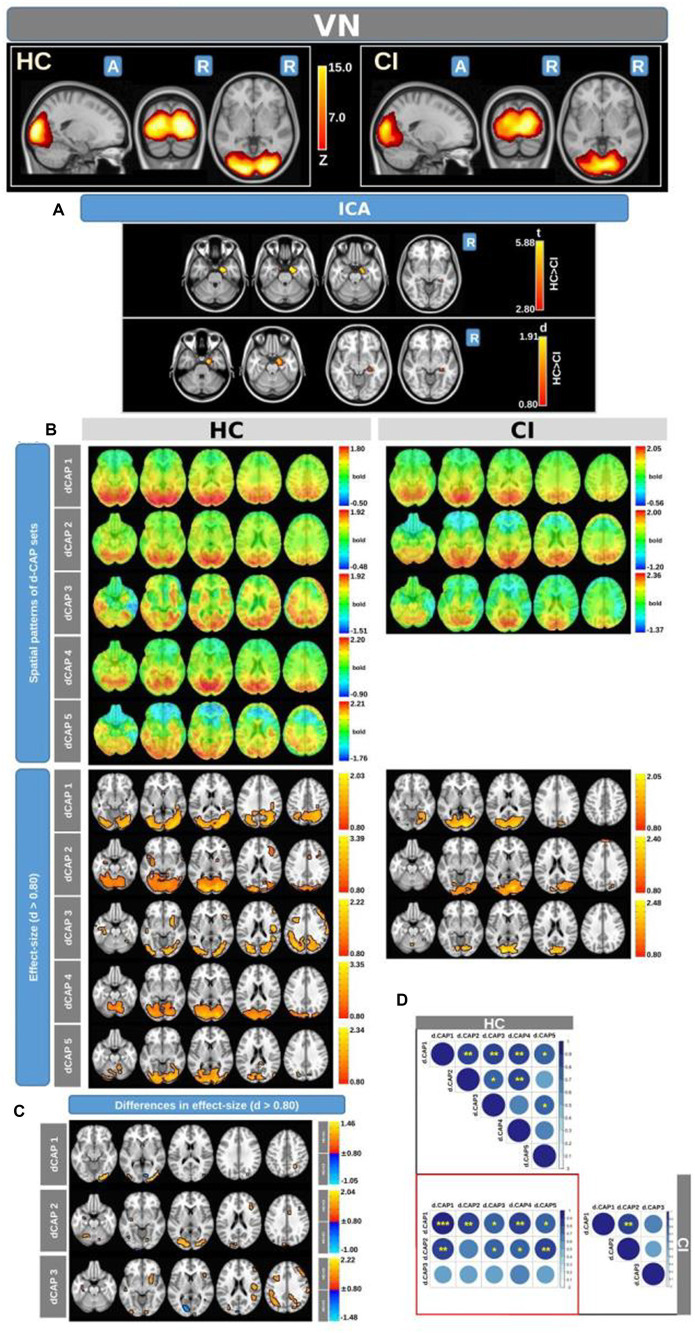
For the VN: Panel **(A)** displays the ICA results for both t-test and effect-size, while panel **(B)** shows the spatial patterns of the d-CAP sets in both the HC and CI groups. In panel **(C)**, effect size maps for each d-CAP in each group are shown. Additionally, panel **(D)** shows the spatial correlation matrices between the d-CAPs, denoted by asterisks (*) to indicate spatial similarities: *for *r* > 0.70, ** for *r* > 0.80, and ***for *r* > 0.90.

Figure 3 displays the Z-values obtained from the dual-regression procedure for the VN. In panel A, the statistical differences between the HC and CI groups are reported for ICA analysis. Significantly lower connectivity was found in the CI group than HC, including in the right hippocampus (<t > =3.367), right parahippocampal gyrus (<t > =3.868), right amygdala (<t > =3.347), and right temporal pole (superior temporal gyrus; <t > =3.199). The complete results, with effect sizes and confidence intervals, are reported in Table 4A.

**Table 4:** Complete results for VN using **(A)** ICA and **(B)** d-CAP analysis.

VN												
(A)												
ICA (t-test clusters)												
	HC > CI (FWE < 0.05 + Bonferroni)											
AAL	Vol (%)	t	*d*	Conf. I								
Hippocampus R	14.89	3.381	1.067	[0.364–1.773]								
ParaHippocampal R	26.21	3.902	1.266	[0.546–1.990]								
Amygdala R	5.11	3.388	1.190	[0.471–1.899]								

(B)												
d-CAPs												
	d-CAP 1				d-CAP 2				d-CAP 3			
	HC > CI		HC < CI		HC > CI		HC < CI		HC > CI		HC < CI	
AAL	Vol (%)	<Δ*d*>	Vol (%)	<Δ*d*>	Vol (%)	<Δ*d*>	Vol (%)	<Δ*d*>	Vol (%)	<Δ*d*>	Vol (%)	<Δ*d*>
Precentral L	-	-	-	-	0.80	1.011	-	-	15.77	1.059	-	-
Precentral R	-	-	-	-	-	-	-	-	6.86	1.117	-	-
Frontal Sup L	-	-	-	-	-	-	-	-	0.96	0.986	-	-
Frontal Sup R	-	-	-	-	-	-	-	-	0.65	1.006	-	-
Frontal Mid L	-	-	-	-	-	-	-	-	1.94	1.028	-	-
Frontal Mid R	-	-	-	-	1.78	0.987	-	-	11.98	1.058	-	-
Frontal Inf Oper L	-	-	-	-	-	-	-	-	2.15	1.091	-	-
Frontal Inf Oper R	-	-	-	-	1.75	0.983	-	-	7.58	0.997	-	-
Frontal Inf Tri R	-	-	-	-	6.21	0.978	-	-	7.48	1.023	-	-
Rolandic Oper R	-	-	-	-	-	-	-	-	20.71	1.090	-	-
Insula R	-	-	-	-	-	-	-	-	3.84	1.003	-	-
Cingulum Mid L	-	-	-	-	-	-	-	-	2.39	0.998	-	-
Cingulum Mid R	-	-	-	-	1.55	0.993	-	-	5.38	1.056	-	-
Cingulum Post R	-	-	-	-	-	-	-	-	0.45	0.982	-	-
Hippocampus L	-	-	-	-	11.88	1.027	-	-	0.66	1.052	-	-
ParaHippocampal L	-	-	-	-	2.40	1.012	-	-	0.93	1.233	-	-
Amygdala L	-	-	-	-	1.90	0.966	-	-	-	-	-	-
Amygdala R	-	-	-	-	-	-	-	-	10.53	1.009	-	-
Calcarine L	-	-	-	-	8.50	1.247	0.28	−0.946	-	-	8.69	−1.133
Calcarine R	4.86	1.022	1.91	−0.928	2.97	1.137	-	-	3.54	1.079	3.80	−1.085
Cuneus L	-	-	-	-	9.52	1.154	-	-	-	-	1.59	−0.994
Cuneus R	1.06	1.002	-	-	4.14	1.159	-	-	1.03	1.031	1.18	−1.060
Lingual L	-	-	-	-	-	-	-	-	2.11	0.998	3.66	−1.114
Lingual R	4.70	0.969	0.11	−0.893	3.26	0.996	-	-	1.30	1.018	-	-
Occipital Sup L	-	-	-	-	11.07	1.158	-	-	2.06	1.005	1.34	−1.100
Occipital Sup R	3.91	0.982	-	-	1.55	1.263	-	-	9.55	1.011	-	-
Occipital Mid L	2.38	0.946	-	-	5.48	1.112	-	-	6.18	1.103	-	-
Occipital Mid R	16.64	1.006	-	-	6.00	1.132	-	-	4.93	0.987	-	-
Occipital Inf L	4.02	0.978	-	-	2.83	1.022	0.77	−0.951	6.80	1.046	-	-
Occipital Inf R	36.97	0.953	-	-	-	-	-	-	-	-	-	-
Fusiform L	-	-	-	-	3.14	1.005	-	-	1.43	1.109	-	-
Postcentral L	-	-	-	-	3.41	1.001	-	-	21.10	1.236	-	-
Postcentral R	-	-	-	-	-	-	-	-	3.51	1.060	-	-
Parietal Sup L	-	-	-	-	13.79	1.006	-	-	22.99	1.163	-	-
Parietal Sup R	-	-	-	-	0.34	0.966	-	-	11.93	1.063	12.20	−1.049
Parietal Inf L	-	-	-	-	4.36	1.028	-	-	30.12	1.097	-	-
Parietal Inf R	-	-	-	-	-	-	-	-	4.30	1.121	0.39	−0.992
SupraMarginal L	-	-	-	-	-	-	-	-	5.82	1.005	-	-
SupraMarginal R	-	-	-	-	-	-	-	-	7.64	1.112	-	-
Angular L	-	-	-	-	2.75	0.974	-	-	22.50	1.140	-	-
Angular R	1.77	0.916	-	-	-	-	-	-	9.14	0.993	1.94	−1.012
Precuneus L	-	-	-	-	2.77	0.985	-	-	23.34	1.084	-	-
Precuneus R	-	-	-	-	4.92	1.027	-	-	27.05	1.147	-	-
Paracentral Lobule L	-	-	-	-	-	-	-	-	22.11	1.069	-	-
Paracentral Lobule R	-	-	-	-	1.94	1.051	-	-	6.01	1.025	-	-
Putamen L	-	-	-	-	0.53	0.953	-	-	-	-	-	-
Putamen R	-	-	-	-	-	-	-	-	5.71	1.081	-	-
Pallidum R	-	-	-	-	-	-	-	-	1.74	1.047	-	-
Temporal Sup L	-	-	-	-	-	-	-	-	2.33	1.005	-	-
Temporal Sup R	-	-	-	-	-	-	-	-	1.07	1.038	-	-
Temporal Mid L	-	-	-	-	-	-	-	-	3.33	1.012	-	-
Temporal Mid R	-	-	-	-	-	-	-	-	3.44	1.038	-	-

AAL, automated anatomical labeling atlas; Vol (%), volume in percent covered from the cluster in the relative AAL area; d, Cohen-d effect-size; Conf.I, confidence interval for the effect-size; <Δ*d*>, differences in effect-size (thresholded at ±0.80).

Panel B displays the spatial patterns of the BOLD signal and the thresholded effect-sizes for the final d-CAP sets for this network. In this case, 5 d-CAPs for HC and only 3 d-CAPs for CI were found. For each d-CAP, between-group differences in Cohen’s *d* maps at *d* ≥ ±0.80 are reported in panel C and Table 4B. Differences in coactivation (mainly with HC > CI) were found in several brain regions (Table 4B). Spatial similarities between d-CAPs are reported in panel D. For this network, high spatial similarities were found across the d-CAPs.

### 3.3 Executive control network

**Figure 4:**
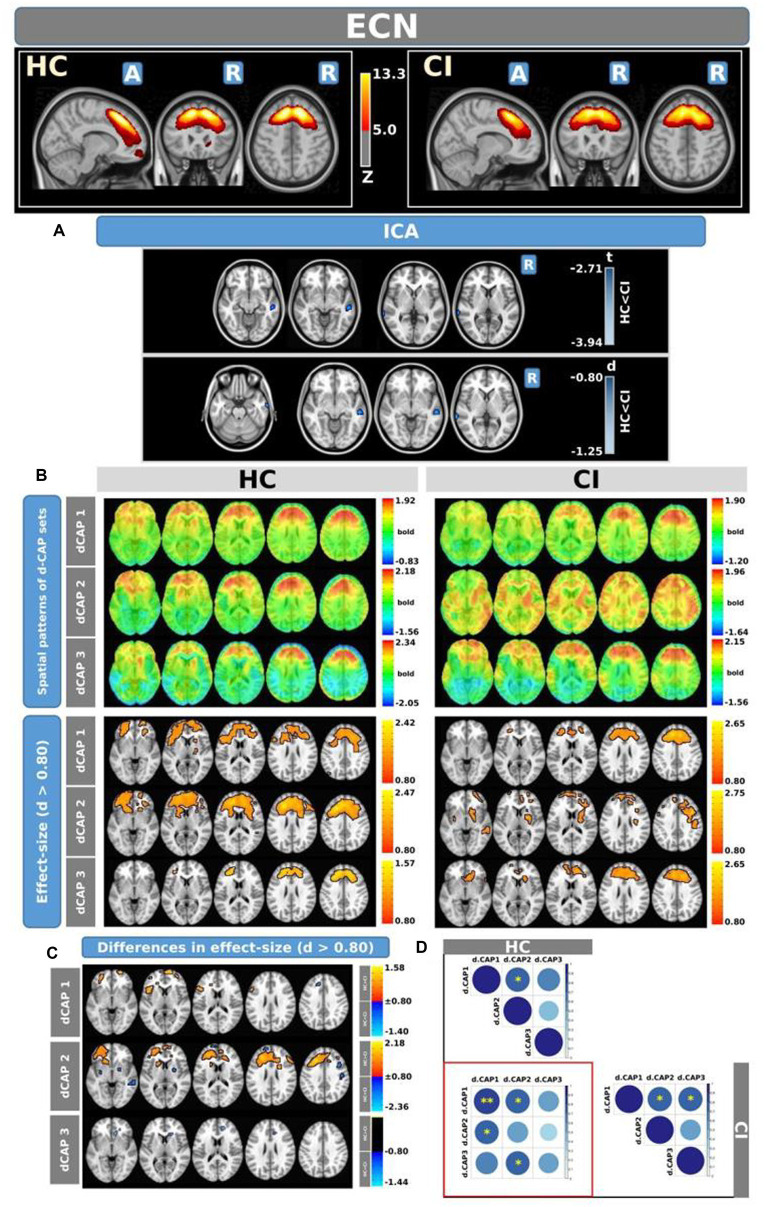
For the ECN: Panel **(A)** displays the ICA results for both t-test and effect-size, while panel **(B)** shows the spatial patterns of the d-CAP sets in both the HC and CI groups. In panel **(C)**, effect size maps for each d-CAP in each group are shown. Additionally, panel **(D)** shows the spatial correlation matrices between the d-CAPs, denoted by asterisks (*) to indicate spatial similarities: *for *r* > 0.70, **for *r* > 0.80, and ***for *r* > 0.90.

Figure 4 shows the Z-values resulting from the dual-regression procedure for the ECN. Panel A focuses on the statistical differences observed between the HC and CI groups in ICA analysis. Notably, a significant decrease in connectivity was discovered within the right superior temporal gyrus (<t > = − 3.177) and both sides of the middle temporal gyrus (<t > = − 3.140) in the HC group compared to the CI group. For comprehensive findings, including effect sizes and confidence intervals, please refer to Table 5A.

**Table 5:** Complete results for ECN using **(A)** ICA and **(B)** d-CAP analysis.

ECN										
(A)										
ICA (t-test clusters)										
	HC < CI (FWE < 0.05 + Bonferroni)									
AAL	Vol (%)	t	*d*	Conf. I						
Temporal Sup R	0.91	−3.180	−1.077	[−1.79–0.36]						
Temporal Mid R/L	1.07	−3.142	−1.023	[−1.72–0.32]						

(B)										
d-CAPs										
	d-CAP 1				d-CAP 2				d-CAP 3	
	HC > CI		HC < CI		HC > CI		HC < CI		HC < CI	
AAL	Vol (%)	<Δ*d*>	Vol (%)	<Δ*d*>	Vol (%)	<Δ*d*>	Vol (%)	<Δ*d*>	Vol (%)	<Δ*d*>
Precentral L	5.95	1.047	-	-	3.34	0.974	4.11	−0.986	-	-
Precentral R	3.37	1.104	-	-	6.67	1.079	-	-	-	-
Frontal Sup L	1.45	0.971	1.75	−1.087	13.75	1.246	3.31	−1.037	1.18	−1.034
Frontal Sup R	5.82	1.121	-	-	3.31	1.077	-	-	3.79	−1.254
Frontal Sup Orb L	7.97	0.970	-	-	18.22	1.019	-	-	-	-
Frontal Sup Orb R	8.28	1.126	-	-	-	-	-	-	-	-
Frontal Mid L	0.99	0.942	-	-	19.37	1.304	1.91	−0.988	0.81	−1.163
Frontal Mid R	3.23	1.070	-	-	2.84	1.028	0.35	−1.087	0.71	−1.145
Frontal Mid Orb L	5.11	0.946	-	-	19.26	0.996	-	-	-	-
Frontal Inf Oper L	13.97	1.018	-	-	3.48	1.196	-	-	-	-
Frontal Inf Oper R	-	-	-	-	2.31	0.961	3.39	−1.235	-	-
Frontal Inf Tri L	9.89	1.019	-	-	11.54	1.006	-	-	-	-
Frontal Inf Tri R	-	-	-	-	6.13	1.024	-	-	-	-
Frontal Inf Orb L	0.54	0.929	-	-	11.28	1.019	-	-	-	-
Supp Motor Area L	0.76	1.062	0.90	−1.053	0.52	0.970	-	-	-	-
Supp Motor Area R	2.83	1.045	-	-	0.25	0.950	-	-	0.96	−1.167
Olfactory L	-	-	-	-	2.83	0.988	-	-	-	-
Frontal Sup Medial L	1.52	0.980	2.32	−1.117	16.36	1.148	1.87	−1.159	1.59	−1.111
Frontal Sup Medial R	7.66	1.112	-	-	5.30	1.067	-	-	0.70	−1.051
Frontal Med Orb L	0.49	0.952	-	-	2.79	0.960	-	-	-	-
Frontal Med Orb R	0.41	1.024	-	-	-	-	-	-	-	-
Rectus L	-	-	-	-	1.31	0.996	-	-	-	-
Insula L	6.42	0.996	-	-	-	-	-	-	-	-
Cingulum Ant L	-	-	-	-	28.04	1.130	-	-	-	-
Cingulum Ant R	-	-	-	-	21.90	1.003	-	-	-	-
Cingulum Mid L	-	-	-	-	3.98	1.133	-	-	-	-
Cingulum Mid R	-	-	-	-	5.50	1.034	-	-	-	-
Hippocampus L	-	-	-	-	-	-	6.67	−1.019	-	-
Hippocampus R	-	-	-	-	-	-	0.29	−1.249	-	-
Amygdala L	-	-	-	-	-	-	0.63	−0.934	-	-
Fusiform R	-	-	-	-	-	-	2.19	−0.999	-	-
Postcentral L	3.23	1.019	-	-	-	-	5.05	−1.062	-	-
Postcentral R	2.89	1.007	-	-	0.54	1.009	1.15	−0.979	-	-
Parietal Inf L	-	-	-	-	-	-	0.46	−1.045	-	-
Precuneus R	0.60	0.974	-	-	-	-	-	-	-	-
Paracentral Lobule R	5.18	0.978	-	-	-	-	-	-	-	-
Caudate L	-	-	-	-	14.35	0.999	-	-	-	-
Caudate R	-	-	-	-	1.36	0.976	-	-	3.56	−0.938
Putamen L	-	-	-	-	10.50	0.975	1.94	−1.111	-	-
Putamen R	-	-	-	-	-	-	4.24	−1.065	-	-
Pallidum R	-	-	-	-	-	-	23.86	−1.096	-	-
Temporal Mid L	-	-	-	-	0.31	0.916	-	-	-	-
Temporal Mid R	-	-	-	-	-	-	9.33	−1.193	-	-
Temporal Inf L	-	-	-	-	5.30	0.990	-	-	-	-
Temporal Inf R	-	-	-	-	-	-	6.28	−1.126	-	-

AAL, automated anatomical labeling atlas; Vol (%), volume in percent covered from the cluster in the relative AAL area; d, Cohen-d effect-size; Conf.I, confidence interval for the effect-size; <Δ*d*>, differences in effect-size (thresholded at ±0.80).

Panel B shows the spatial patterns of the BOLD signal and the thresholded effect sizes for the final d-CAP sets for this network. In this case, for both groups, we found 3 d-CAPs. Additionally, for each d-CAP, between-group differences in Cohen’s *d* maps at *d* ≥ ±0.80 are reported in panel C and Table 5B. Differences in coactivation were found in several brain regions. For d-CAP-3, we found only clusters with higher coactivation in CI than HC (Table 5B). Lastly, spatial similarities between d-CAPs are reported in panel D. High spatial similarity was found for d-CAP-1 between HC and CI (r > 0.80).

### 3.4 Frontoparietal network

**Figure 5:**
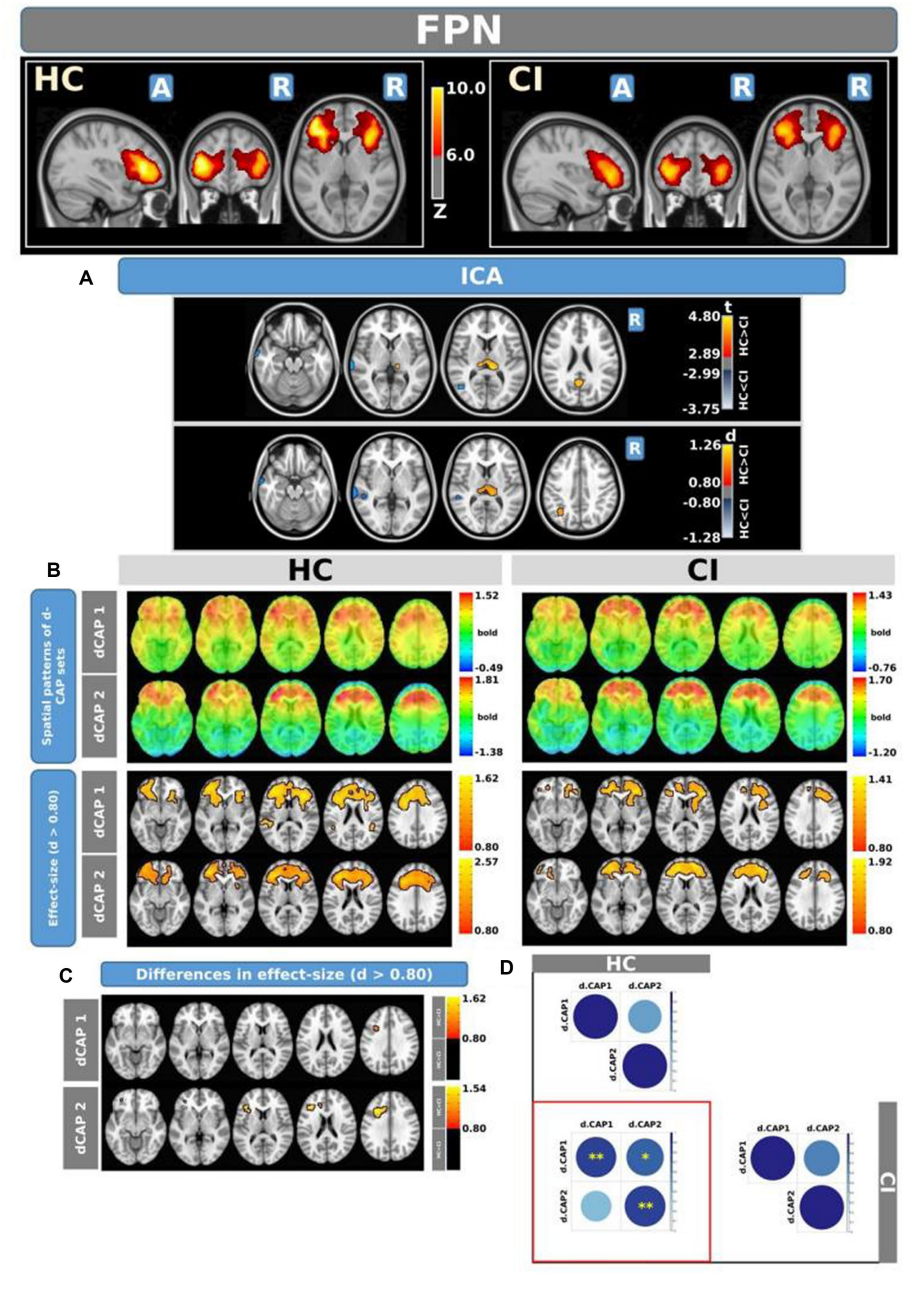
For the FPN: Panel **(A)** displays the ICA results for both t-test and effect-size, while panel **(B)** shows the spatial patterns of the d-CAP sets in both the HC and CI groups. In panel **(C)**, effect size maps for each d-CAP in each group are shown. Additionally, panel **(D)** shows the spatial correlation matrices between the d-CAPs, denoted by asterisks (*) to indicate spatial similarities: *for *r* > 0.70, **for *r* > 0.80, and ***for *r* > 0.90.

Figure 5 shows the Z-values resulting from the dual-regression procedure for the FPN. Panel A shows the statistical differences observed between the HC and CI groups in ICA analysis. Compared with CI, significant clusters with higher connectivity were observed in HC, mainly in the left posterior cingulate gyrus (<t > =3.055), left superior parietal gyrus (<t > =3.068), precuneus (<t > =3.141), and thalamus (<t > =3.439 and < t > =3.198, for left and right sides, respectively). A small cluster with higher connectivity in CI was found inside the left inferior frontal gyrus pars orbitalis (<t > = − 3.103). For comprehensive findings, including effect sizes and confidence intervals, please refer to Table 6A.

**Table 6:** Complete results for FPN using **(A)** ICA and **(B)** d-CAP analysis.

FPN				
(A)				
*ICA (t-test clusters)*				
	HC > CI (FWE < 0.05 + Bonferroni)			
AAL	Vol (%)	t	*d*	Conf. I
Cingulum Post L	1.54	3.058	0.820	[0.133–1.509]
Parietal Sup L	1.60	3.108	0.845	[0.151–1.521]
Precuneus L/R	2.14	3.146	0.756	[0.072–1.434]
Thalamus L	13.04	3.431	1.081	[0.371–1.788]
Thalamus R	4.14	3.206	1.024	[0.322–1.721]
	HC < CI (FWE < 0.05 + Bonferroni)			
AAL	Vol (%)	t	*d*	Conf. I
Frontal Inf Orb L	3.64	−3.106	−0.888	[−1.571–0.192]
(B)				
*d-CAPs*				
	d-CAP 1		d-CAP 2	
	HC > CI		HC > CI	
AAL	Vol (%)	<Δ*d*>	Vol (%)	<Δ*d*>
Frontal Mid L	-	-	0.94	1.094
Frontal Mid Orb L	-	-	3.12	0.989
Frontal Inf Oper L	1.60	0.984	1.90	1.032
Frontal Inf Oper R	-	-	0.37	0.941
Frontal Inf Tri L	0.21	0.933	7.62	1.076
Supp Motor Area L	-	-	4.49	0.978
Supp Motor Area R	-	-	3.15	0.968
Frontal Sup Medial L	-	-	0.25	0.925
Insula L	-	-	1.41	0.951
Cingulum Ant L	-	-	1.80	0.950
Cingulum Mid L	-	-	4.58	0.995
Cingulum Mid R	-	-	3.88	0.961

AAL, automated anatomical labeling atlas; Vol (%), volume in percent covered from the cluster in the relative AAL area; d, Cohen-d effect-size; Conf.I, confidence interval for the effect-size; <Δ*d*>, differences in effect-size (thresholded at ±0.80).

Panel B shows the spatial patterns of the BOLD signal and the thresholded effect sizes for the final d-CAP sets for this network. For this network, for both groups, we found 2 d-CAPs. Moreover, for each d-CAP, between-group differences in Cohen’s *d* maps at *d* ≥ ±0.80 are reported in panel C and Table 6A. Compared with the CI group, higher coactivation was found in HC inside several brain regions (Table 6B). Lastly, spatial similarities between d-CAPs are reported in panel D. High spatial similarity was found for d-CAP-1 between HC and CI (*r* > 0.80).

### 3.5 Switching probability, spatial consistency, and temporal fraction

**Figure 6:**
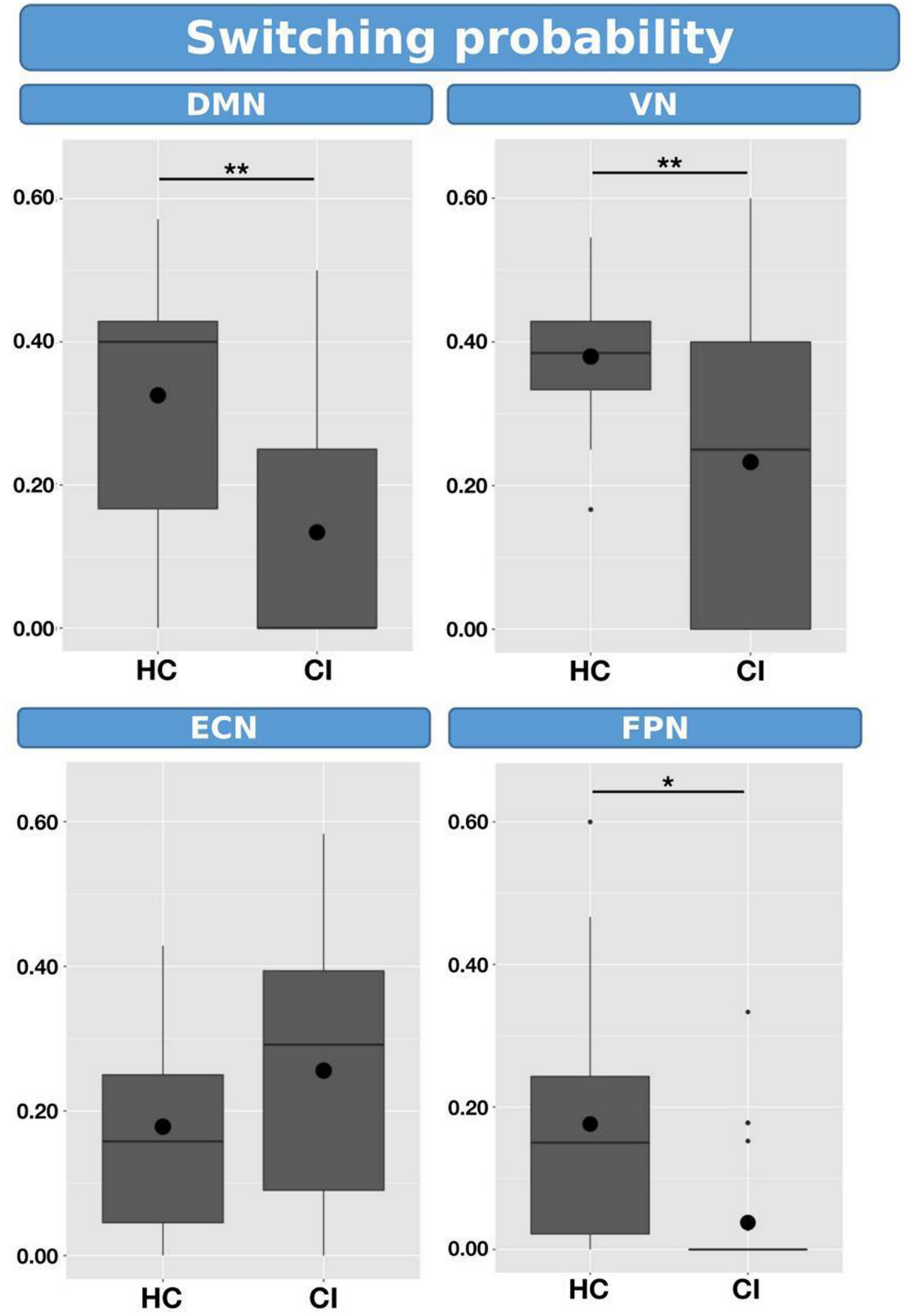
Box plots illustrating the switching probabilities of d-CAPs for the HC and CI groups. Statistical significance was denoted by *for FDR-values less than 0.05 and **for FDR-values less than 0.001.

Switching probabilities were computed for each participant within the HC and CI groups. Box plots illustrating the switching probabilities associated with all networks are depicted in Figure 6, while the complete results can be found in Table 7. Notably, when comparing the CI group to the HC group, significantly lower switching probabilities were observed for the DMN (*t* = 2.239; FDR = 0.023), VN (*t* = 2.864; FDR = 0.007), and FPN (*t* = 2.645; FDR = 0.017). However, for the ECN, although a decrease in switching probabilities was observed in the HC group, the corresponding FDR-value was greater than 0.05 (*t* = 0.461; FDR = 0.764). The decreased switching probabilities in the CI group, particularly for the DMN, VN, and FPN networks, indicate an overall reduction in dynamic activity within these networks.

**Table 7:** Statistical comparisons were conducted to compare the switching probability between the HC and CI groups.

	<HC>	<CI>	t	*FDR*
DMN	0.37 (0.21)	0.19 (0.26)	2.239	0.023*
VN	0.38 (0.10)	0.23 (0.21)	2.864	0.007**
ECN	0.19 (0.25)	0.26 (0.20)	0.461	0.764
FPN	0.18 (0.19)	0.04 (0.08)	2.645	0.017*

The mean and standard deviation of the switching probability for each network are provided. Additionally, network-specific t-values and FDR-values are presented.

Table 8A presents the spatial consistency of each d-CAP associated with all networks. Across the analyzed networks, the mean spatial consistency for the first d-CAP was higher in the HC group compared to the CI group, which can indicate resting-state networks with reduced dynamics in the latter.

**Table 8:** **(A)** Spatial consistency for all networks; **(B)** Temporal fraction of each d-CAP associated with the four networks in HC and CI groups; **(C)** Statistical comparisons of the temporal fraction of the 1st d-CAP associated with networks that share the same number of d-CAPs in the HC and CI groups.

(A) Consistency								
	HC					CI		
	d-CAP 1	d-CAP 2	d-CAP 3	d-CAP 4	d-CAP 5	d-CAP 1	d-CAP 2	d-CAP 3
DMN	0.72 (0.11)	0.67 (0.11)	0.60 (0.21)			0.71 (0.10)	0.62 (0.11)	0.75 (0.12)
VN	0.75 (0.10)	0.61 (0.11)	0.76 (0.08)	0.63 (0.10)	0.75 (0.12)	0.68 (0.14)	0.67 (0.10)	0.59 (0.10)
ECN	0.75 (0.15)	0.66 (0.10)	0.69 (0.15)			0.58 (0.13)	0.70 (0.15)	0.62 (0.14)
FPN	0.62 (0.15)	0.56 (0.12)				0.56 (0.11)	0.54 (0.11)	
**(B) Temporal fraction**								
	HC		CI					
	d-CAP	TF (%)	dCAP	TF (%)				
DMN	d-CAP 1	0.52	d-CAP 1	0.69				
	d-CAP 2	0.15	d-CAP 2	0.21				
	d-CAP 3	0.16	d-CAP 3	0.14				
VN	d-CAP 1	0.34	d-CAP 1	0.53				
	d-CAP 2	0.21	d-CAP 2	0.27				
	d-CAP 3	0.14	d-CAP 3	0.20				
	d-CAP 4	0.16						
	d-CAP 5	0.14						
ECN	d-CAP 1	0.39	d-CAP 1	0.41				
	d-CAP 2	0.22	d-CAP 2	0.28				
	d-CAP 3	0.39	d-CAP 3	0.30				
FPN	d-CAP 1	0.61	d-CAP 1	0.73				
	d-CAP 2	0.27	d-CAP 2	0.39				
**(C) Temporal fraction for 1st d-CAP**								
	HC	CI	t	FDR				
DMN	0.52 (0.14)	0.69 (0.11)	−4.180	0.0006**				
ECN	0.39 (0.12)	0.42 (0.12)	−0.751	0.4562				
FPN	0.61 (0.15)	0.73 (0.12)	−2.729	0.0147*				

In **(C)**, *indicates FDR < 0.05 and **indicates FDR < 0.01.

In Table 8B, the temporal fraction of the d-CAPs associated with each network is shown. Additionally, Table 8C provides the mean and SD of the temporal fraction for the 1st d-CAPs in each group for only networks with the same number of d-CAPs. Statistical differences were found in the DMN and FPN networks, where the HC group exhibited lower temporal fractions compared to the CI group, indicative of decreased network dynamics for the CI group.

### 3.6 Correlations

**Figure 7:**
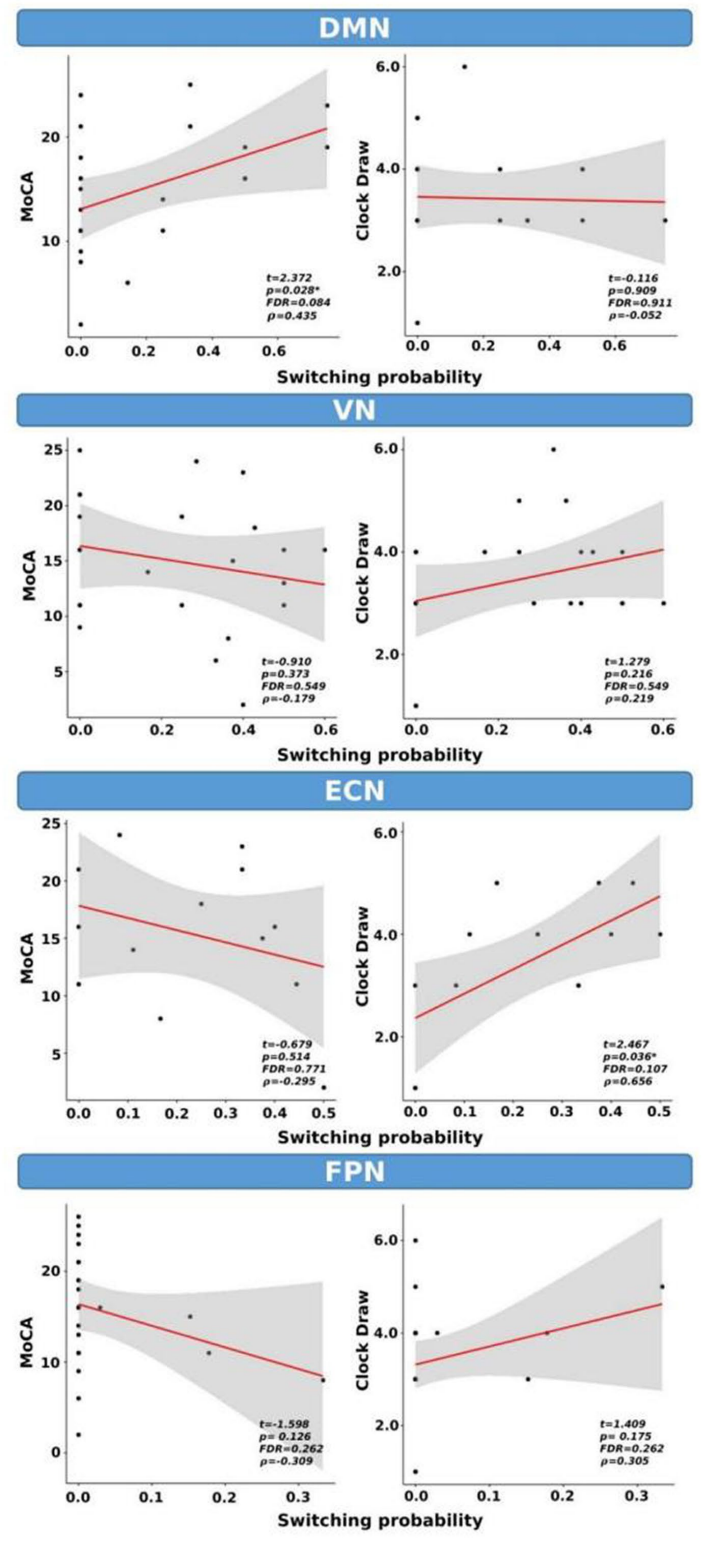
Switching probability as a function of cognitive scores for CI group only. *indicates *p* < 0.05 (or FDR < 0.05). ρ: Spearman’s rank correlation coefficient.

We examined the correlations between switching probability and cognitive scores, as shown in Figure 7. Our analysis revealed statistical correlations, without correction for multiple comparisons, between MoCA scores and SP within the DMN (*t* = 2.372, *p* = 0.028, FDR = 0.084, ρ = 0.43). Similarly, a significant correlation was observed between the clock draw and SP within the ECN (*t* = 2.467, *p* = 0.036, FDR = 0.107, ρ = 0.656). It is important to note that both correlations did not survive FDR correction.

## 4 Discussion

In this preliminary study, we used ICA and d-CAP analysis methods to investigate the differences in resting-state functional connectivity and dynamic network properties between HC and individuals with CI. Additionally, differences in cognitive performances between the two groups and correlations between cognitive scores and d-CAP switching probability were analyzed.

We found the same numbers of d-CAPs between the HC and CI groups for the DMN, ECN, and FPN. However, the VN exhibited more d-CAPs in the HC group than in the CI group. These results suggest that the dynamic properties of the VN may be altered in individuals with CI.

By ICA analysis, significant differences in connectivity between the HC and CI groups were found in the DMN. Specifically, lower connectivity was observed in the CI group compared to HC in regions such as the right precentral gyrus and right postcentral gyrus. These findings align with previous research indicating altered DMN connectivity in individuals with CI and dementia (Greicius et al., 2004; Sorg et al., 2007; Bai et al., 2009). In addition, spatial maps and effect size analyses revealed increased coactivation in the HC group compared to CI, reflecting more synchronized activity within the DMN in healthy individuals.

The DMN plays a crucial role in several cognitive functions, such as autobiographical memory and social cognition. Alterations in the DMN have been identified as potential early markers of cognitive decline, including the early stages of AD (Grieder et al., 2018). Additionally, as AD progresses, there are disruptions in the connectivity and activity within the DMN. This network is also closely tied to processes related episodic memory, which is often impaired in AD (Huo et al., 2018). The DMN has been previously studied in dementia, providing valuable insights into the pathophysiology of various forms of CI (Sorg et al., 2007). For instance, decreased functional connectivity inside the DMN in patients with AD has been reported by several studies (Greicius et al., 2004; Sorg et al., 2007), and it may be associated with deficits in different cognitive domains, including memory and attention (Hahn et al., 2013).

The VN also showed significant differences in connectivity between the HC and CI groups. The HC group displayed higher connectivity in regions including the hippocampus, parahippocampal gyrus, amygdala, and temporal pole (superior temporal gyrus). From d-CAPs analysis, we found five d-CAPs for HC and only three d-CAPs for CI, which might indicate a less dynamic network inside people with CI.

The VN is a network essential for processing visual information and connects to cognitive functions like object recognition and spatial awareness (Uddin et al., 2010). Changes in the VN have been associated with cognitive decline and visual impairments, which can occur in dementia, leading to challenges in tasks such as object recognition, spatial orientation, and visual attention (Frost et al., 2013); additionally, changes in the visual system might serve as early indicators of cognitive decline (Marquié et al., 2019; Xia et al., 2022). Furthermore, studies have shown that VN alterations are associated with specific visual symptoms in AD (Huang et al., 2021). For instance, decreased connectivity between VN regions has been correlated with deficits in visual attention and memory (Lazarou et al., 2022).

Similarly, the FPN showed lower connectivity in CI than HC in regions such as the left posterior cingulate gyrus, left superior parietal gyrus, precuneus, and thalamus. The FPN is of significant interest in the AD due to its involvement in executive functions, attentional control, and cognitive flexibility (Agosta et al., 2013). Several studies have demonstrated changes in FPN connectivity in individuals with dementia, which might correspond to the underlying cognitive impairments (Zhou et al., 2010).

Various cognitive processes such as attention control, working memory, cognitive flexibility, and goal-directed behavior are associated with the ECN (Dajani and Uddin, 2015). In this study, the ECN showed decreased connectivity in the HC group compared to CI, particularly within the right temporal regions. Although higher functional connectivity inside the ECN is not common in individuals with dementia, Liu et al. similarly found increased connectivity in a cohort of individuals with MCI (Balachandar et al., 2015; Liu W. et al., 2021).

For all networks, the spatial similarity, which reflects the spatial correlations between each network-associated time frame and every d-CAP, showed a good correspondence between HC and CI in several d-CAPs.

From d-CAPs analysis, we found that the switching probabilities were significantly reduced in the DMN, VN, and FPN networks in individuals with CI. These findings suggest reduced dynamics and less flexible functional interactions within these networks in CI. However, no significant differences in switching probability were observed for the ECN, indicating preserved dynamic properties in this network between the HC and CI groups. Additionally, the spatial consistency of d-CAPs showed higher values in the HC group than the CI group across all networks, indicating greater consistency in the functional connectivity patterns within d-CAPs in healthy individuals.

The temporal fraction for the 1st d-CAP revealed statistical differences in the DMN and FPN networks. The CI group exhibited higher temporal fractions compared to HC. These findings suggest that the DMN and FPN in CI are less dynamic networks.

Finally, the correlations between SP and cognitive scores for the CI group showed significant statistical correlations between the MoCA scores and SP within the DMN. Similarly, a significant correlation was observed between clock draw performance and SP within the ECN (*p* < 0.05). However, these correlations did not survive after multiple comparison corrections.

In this investigation, distinct ICA analyses were conducted for each group, and the multi-session temporal concatenation option was employed. This methodology was chosen to capture unique patterns of functional connectivity specific to each cohort, facilitating a more nuanced exploration of group-specific alterations. Moreover, given that the primary aim of our study was to analyze differences in resting-state functional connectivity and dynamic network properties using dCAPs, this approach was considered the most fitting for our specific research objectives.

One limitation of this study is the relatively small sample size; therefore, we have defined this study as “preliminary.” This designation reflects our awareness of the exploratory nature of the research and the need for caution in interpreting the results. For this reason, effect sizes have been reported for all analyses, which measure the strength of a relationship between variables or the magnitude of an intervention’s impact. In studies with small sample sizes, detecting meaningful effect sizes becomes challenging. Additionally, the CI cohorts were defined based on clinical diagnosis, without biomarker confirmation (Jack et al., 2018). Therefore, these findings should be replicated in a larger cohort, with biomarker confirmation (Abubakar et al., 2012), to enhance the robustness of the results and facilitate a more comprehensive understanding of the condition.

Another limitation of our study lies in the unavailability of respiratory and cardiac data during the MRI acquisition. Despite recognizing the importance of addressing potential artifacts from physiological sources such as the respiratory and cardiac systems, we were constrained by the absence of the capability to acquire specific data related to these variables. Although we have incorporated this limitation into our discussion, the absence of respiratory and cardiac data during the imaging process might pose a restriction on the fMRI analysis.

Finally, other limitations are associated with the proposed CAP group analysis. First, using k-means clustering with spatial correlation as a similarity measure may not fully capture the non-linear relationships between different time frames in fMRI data. Consideration of alternative clustering methods that can account for non-linear relationships might be useful to enhance the accuracy and efficiency of the clustering process (Liu B. et al., 2021; Zou et al., 2021). Additionally, direct comparisons between d-CAPs from different groups are challenging due to the group-specific nature of network dynamics represented by d-CAP sets. As a result, except for the first d-CAP, the correspondence of all d-CAPs between groups cannot be maintained (Zhuang et al., 2018). Consequently, comparisons involving specific d-CAPs other than d-CAP1 cannot be directly performed. Considering the inherent interdependence between the number of d-CAPs, it is important to interpret results for group comparisons by using temporal fractions and switching probabilities. Therefore, addressing these limitations and incorporating more sophisticated clustering techniques could enhance the robustness and interpretability of the proposed CAP group analysis method.

## 5 Conclusion

In conclusion, our results provide evidence for altered resting-state functional connectivity and dynamic network properties in individuals with CI compared to HCs. The findings suggest altered connectivity patterns within the DMN, VN, ECN, and FPN, indicating the involvement of multiple functional networks in CI. Our results revealed reduced connectivity in the DMN, VN, and FPN, while showing increased connectivity in the ECN, possibly indicative of compensatory processes.

Additionally, decreased switching probabilities and higher temporal fractions highlight the diminished dynamics and flexibility of functional interactions within these networks in people with CI. These results can help to understand the brain processes underlying CI and influence the development of diagnostic and therapeutic strategies specifically targeting these networks.

## Data availability statement

The datasets presented in this article are not readily available because privacy. Requests to access the datasets should be directed to Ashley.Stokes@barrowneuro.org.

## Ethics statement

The studies involving humans were approved by Dignity Health Institutional Review Board. The studies were conducted in accordance with the local legislation and institutional requirements. The participants provided their written informed consent to participate in this study.

## Author contributions

MB: Writing – review & editing, Writing – original draft, Software, Methodology, Investigation, Formal analysis, Data curation, Conceptualization. AB: Writing – review & editing, Validation. MS: Writing – review & editing, Validation. RC: Writing – review & editing, Validation. LB: Writing – review & editing. AS: Writing – review & editing, Writing – original draft, Visualization, Validation, Supervision, Funding acquisition.

## Footnotes

^1^ https://github.com/rordenlab/dcm2niix

^2^ https://afni.nimh.nih.gov/

^3^ https://fsl.fmrib.ox.ac.uk/fsl/fslwiki/FSL
